# Perceptions of colorectal cancer screening in the Arab American community: a pilot study

**DOI:** 10.1017/S1463423619000161

**Published:** 2019-07-01

**Authors:** Muhammad Alsayid, Nour Mazen Tlimat, Clarence Spigner, Christian Dimaano

**Affiliations:** 1 Department of Health Services, School of Public Health, University of Washington, Seattle, WA, USA; 2 University of Massachusetts Memorial Medical Center, University Campus, Worcester, MA, USA; 3 Department of Medicine, Oak Hill Hospital, Brooksville, FL, USA

**Keywords:** Arab American, barriers, colorectal cancer screening, facilitators, qualitative study

## Abstract

**Objective:**

Multiple factors such as socioeconomic status (SES), education, race, and ethnicity can affect colorectal cancer screening (CRCS) rates. However, few studies have addressed CRCS disparities among Arab Americans. Our aim was to understand how Arab Americans view CRCS.

**Method:**

Employing thematic analysis, we collected and analyzed the dialogue of Arab American focus groups and interviews to better understand participants’ perceptions of CRCS. Themes were generated and categorized into barriers and facilitators.

**Results:**

Eleven Arab American males participated in two focus groups and two interviews. Three barriers included disbelief in modern medicine, concerns about the procedure, and lack of communication with the physician. Three facilitators were also identified: compliance and priority of health, access to healthcare, and awareness.

**Conclusion:**

Disparities in CRCS cannot solely be explained by SES and access but cultural differences also contribute. Specific interventions accounting for these cultural differences are needed to reduce disparities in CRCS among Arab Americans.

## Introduction

Colorectal cancer (CRC) is the second leading cause of cancer-related deaths in the USA. Although there is strong evidence supporting the benefits of colorectal cancer screening (CRCS), the rates for either stool-based tests or endoscopy in 2015 reached only 62.6% – lower than the goal set by the National Colorectal Cancer Roundtable (NCCRT) (80% by 2018) (American Cancer Society, 2018). Factors like socioeconomic status (SES), education, health insurance, and income can affect CRCS rates in certain at-risk populations. In particular, race and ethnicity play key roles in CRCS (Liss and Baker, 2014). While lack of insurance and access to healthcare are associated with low CRCS rates, they are not the only factors among minority groups (Stimpson *et al*., 2012). Lack of CRC knowledge, fear and embarrassment of the procedure, physician recommendation, low health literacy, and fear of screening outcomes comprise additional barriers (Hennelly *et al*., 2015; Miranda-Diaz *et al*., 2015).

## Purpose

The Arab American Institute Foundation estimated that nearly 3.7 million Americans are of Arab descent (American Arab Institute, 2018). According to a self-reported survey in 2013, the rate of CRCS in Arab Americans aged 50 years and older living in Michigan was only 48.5% – significantly lower than the CRCS rate of all Michigan adults in the same age group (MI Department of Health and Human Services, 2018). This indicates a disparity in screening rates within Arab Americans compared to other racial and ethnic groups (Michigan Department of Health and Human Services, 2018). Understanding the barriers and facilitators to CRCS in a specific ethnic group is the first step toward the creation of culturally appropriate interventions. However, insufficient data exist regarding Arab Americans’ beliefs and attitudes toward CRCS (Talaat, 2015). Here, we explored the views and attitudes of Arab Americans toward CRCS, using qualitative methods to interrogate barriers and facilitators. Understanding the complexities of how Arab Americans may be at risk for screening disparities can help to inform primary care physician strategy in approaching this population of patients for intervention.

## Methods

### Conceptual framework

**Figure 1.**
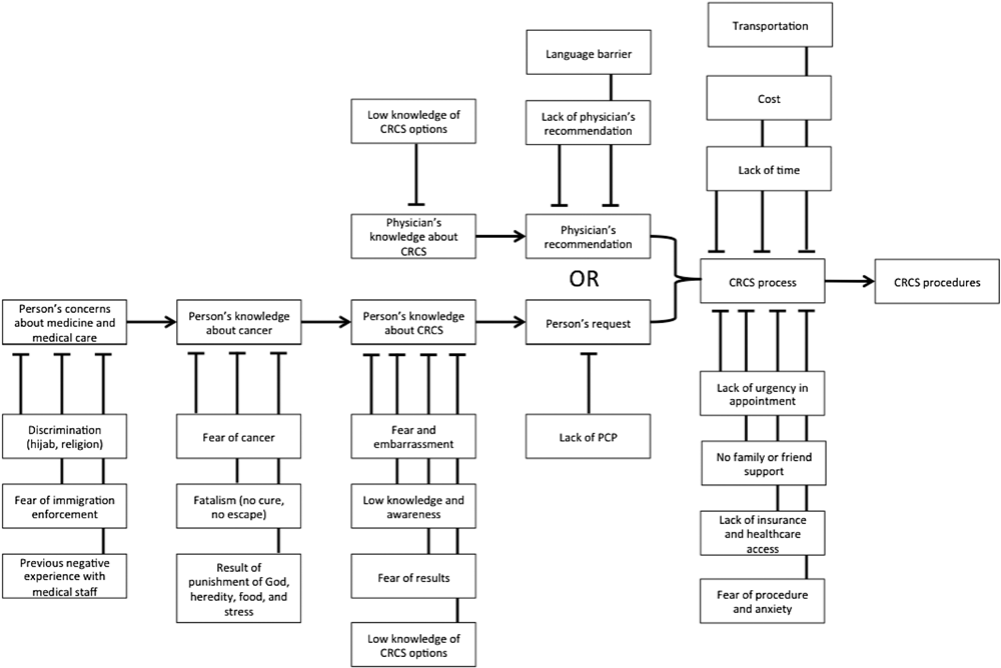
Conceptual framework of CRCS in Arab Americans

In order to understand the process of CRCS and the potential perceived barriers within the Arab American community, we built a conceptual framework outlining how complex factors might interact, overlap, and feed into a person’s hesitancy to being screened for CRC (Figure 1). Since the factors affecting decision-making on CRCS is multifactorial, this conceptual framework helped to identify key topics of interest which we focused on in the development of our interview questions.

### Participants

Arab Americans living in the San Francisco Bay Area of California and Worcester, Massachusetts, participated in the study. We targeted participants who met the following eligibility criteria for study recruitment: 50–75 years old, born in an Arab country, and identified themselves to be of Arab ethnicity. People with active or prior history of CRC were excluded. Tactics to obtain study participation included public announcements of the study at Arab-majority mosques and flyer placement in Mediterranean stores. Eleven Arab Americans participated in two focus groups and two individual interviews; the first group was made up of six participants and the second group had three participants.

### Discussion questions

Discussion questions were developed to assess the participants’ perceptions of barriers and facilitators to CRCS (Appendix 1). These questions were not taken from a validated instrument as the intention of this pilot study was hypothesis generating. However, questions were intentionally left broad to elicit frank responses. In addition, more detailed probing questions were also asked based on the participants’ specific experience with CRCS.

### Data collection

Following explanation of the study, written informed consent from each participant was obtained. Each participant also provided general demographic information. The responses were recorded using a digital audio recorder, and two bilingual researchers (interviewer and scribe) transcribed the Arabic commentary into English. Two methods of data collection were used: focus groups or one-on-one interviews. One-on-one interviews were used when only one participant attended the planned focus group. Each focus group lasted approximately 45 minutes and each interview lasted approximately 30 minutes. Each participant received a $25 gift card to compensate for the use of his time (the participants were only made aware of the gift card upon completion of the focus group or interview). At the end of each focus group and interview, a brief presentation about CRCS was given along with brochures and CRC educational materials that were obtained from the American Cancer Society.

### Analysis

Participants were given the choice to speak in English or Arabic. This choice was important to facilitate proper dialogue with those participants who could not describe highly technical medical issues, procedures, or concerns properly in English. The first focus group was conducted in English based on the participants’ preference. The second focus group and subsequent two individual interviews were conducted in Arabic which required translation. Two bilingual researchers (interviewer and scribe) transcribed the Arabic commentary into English. Following translation of transcripts in Arabic to English, transcripts were back-translated to ensure accuracy. The data were then analyzed and transcripts were manually coded without the assistance of computer software. Relevant words, sentences, and phrases were labeled with codes, and a code dictionary was built through several iterations. Coding was done independently by the corresponding authors to ensure inter-rater reliability. We used an iterative process, loosely based on Grounded Theory, to generate themes which were then categorized into barriers and facilitators. This study was approved by the University of Washington’s Institutional Review Board (IRB ID: STUDY00000666).

## Results

Participant recruitment was done through flier advertisements as described above. Demographics of the study participants are listed in Table 1. Seven participants had regular CRCS through colonoscopy and/or fecal immunochemical test (FIT). The mean age of all participants was approximately 63 and all were men. All participants were Muslims and US citizens or legal permanent residents.

**Table 1.** Demographics of study participants

Demographics	Number
Total	11
English level	
Advanced	4
Intermediate	6
Beginner	1
Annual income, USD*	
<$20 000	2
$20 000–$49 000	2
$50 000–$100 000	2
> $100 000	3
Education level	
6–8 years	1
9–12 years	3
Some college/university	1
University graduate	6
Marital status	
Single	2
Married	9
Have a primary care physician	10
Have health insurance	10

* Two participants did not disclose their annual income.

Six themes were identified; three were categorized into barriers, while the other three into facilitators. A list of themes with relevant quotes is shown in Table 2. The barriers included disbelief in modern medicine, concerns about the procedure, and lack of communication with physician. Three facilitators were also identified: compliance and priority of health, access to healthcare, and awareness.

**Table 2.** Themes generated with illustrative participant insights

**(A) Barriers**
I–Disbelief in modern medicine *The word cancer does not mean anything but it is a trick for pharmaceutical companies and chemical industries to sell the illusion that there is something and to sell their medications*
II–Concerns about the procedure *I didn’t want to put anything in my rear and it should have been easier way to do it*.
III–Lack of communication with the physician *Actually, they send the kit and never heard back. I have never seen anything. The only thing, the doctor responds to the email…*
**(B) Facilitators**
I–Compliance and priority of health *Until now, I didn’t refuse any test as long as it is for my benefit…*
II–Access to healthcare services *Kaiser sent everybody. I think after 50. They sent you the stool, the blood in the stool test*.
III–Awareness *I was really aware of colon cancer because my aunt’s husband died of it in Jordan*.

### Disbelief in modern medicine

At least three participants expressed their disbelief in the term ‘cancer’ and none of them had regular CRCS. Some participants preferred lifestyle modification like changing dietary habits and exercise in favor of taking medications or performing further tests. One participant who had irregular visits to the doctor responded to our question about his views of cancer as follows: *…The word cancer does not mean anything but it is a trick for pharmaceutical companies and chemical industries to sell the illusion that there is something and to sell their medications and nuclear medicine and radiation and these chemical substances that has high costs*.

### Concerns about the procedure

Six participants had colonoscopy, in which one described: *It is not fun, I hate it* and *It is painful*. Another mentioned the difficulties he faced throughout the process. *It was not easy…You have to have somebody drives you back. You have to take your wife or a friend and you are under anesthesia…You have to drink a lot of liquid…It is pain in the butt, really. Both ways…it is really something I don’t look forward to*. The majority of them considered the colonoscopy preparation a huge barrier and retorted that an easier procedure should be discovered.

### Lack of communication with the physician

Some participants believed that physicians did not actually provide them with appropriate care. Although they possessed health insurance, participants mentioned that visits with the primary care physician were irregular and brief due to time restrictions on office visits, expressing that communication with the physicians was typically limited. As stated by one participant: *Maybe every five to six months or when there is an emergency. You go to the appointment and you do an interview with your doctor. And I have tried that even when you have an emergency. When you go they do not show that much of a care*.

Participants who never had regular CRCS expressed that their primary care physicians did not provide information about CRC or CRCS methods. One participant stated: *They did not explain to me. They just said we need to do colonoscopy. They did not say why they will do it*.

### Compliance and priority of health

The vast majority of participants believed that complying with the physician’s recommendations is beneficial for their health. One of the participants stated: *Until now, I didn’t refuse any test as long as it is for my benefit. I mean, the doctor when he decides something usually it is based on a specific thing. I mean, it is based on something important. I have to do it*. Another person expressed his beliefs toward preventative measures by saying: *And I am a believer in anything that has a preventive type of action. If it is available for me, I can basically make sure I am healthy from something inside that I do not see*.

### Access to healthcare services

Access to healthcare (ie: participants having health insurance in the USA) is a major factor that introduced most participants to CRCS and allowed them to receive appropriate health information from their physicians. One of the participants who had seen the same primary care physician for 25 years through the healthcare system, Kaiser Permanente, responded to a question about his knowledge of CRC and said: *Kaiser sent everybody. I think after 50. They sent you the stool, the blood in the stool test. They mail it actually to you and you mail it back. This is a very good way that Kaiser keeps it up to front and let every member knows that you need to do this*.

### Awareness

Awareness of CRC as a debilitating disease and the awareness of the need for regular screening directly can facilitate loyal and consistent CRCS. Some participants, who kept up with their own CRCS, expressed their awareness of CRC and CRCS methods through discussions with family and friends, while others learned about it from their primary care physicians. The group generally believed that CRC is preventable and curable if found at an early stage, with one participating stating: *I think if you discover it early you get a better chance of survival and cure*. When asked about where their knowledge of CRC came from, one responded: *Mostly I am educated on colon cancer from my physician*.

## Discussion

Our study explored the perceptions of Arab Americans toward CRCS and identified six key findings categorized as three barriers to screening and three facilitators to screening. The barriers included: (1) a disbelief in modern medicine; (2) concerns about the CRCS procedure; and (3) lack of communication with the physician. Facilitators identified included: (1) priority of health; (2) access to healthcare services; and (3) awareness. Interestingly, the findings of our study also correlate with the results of a systematic review that reported several barriers and facilitators in the general population as well (Honein-AbouHaidar *et al*., 2016). In addition, two previous studies evaluated the barriers to CRCS in Arab Americans through quantitative analysis; however, the strength of our study is that this is, to our knowledge, the first qualitative study examining CRCS specifically among Arab Americans (Talaat and Harb 2013; Talaat, 2015). The barriers reported in these previous studies were lack of awareness, feeling uncomfortable, and lack of physicians’ recommendation. Our study reported similar barriers, but our thematic analysis also identified ‘disbelief in modern medicine’ as a new, additional barrier. Our study showed that participants believed that lifestyle modifications like changing diet and exercise are sufficient to prevent CRC. Disbelief in modern medicine could stem from the healthcare systems in Arab countries and the participants’ cultural upbringing. The majority of healthcare systems in Arab countries do not promote cancer prevention programs and most Arabs do not have experience with preventative services. The concept of preventive medicine is largely lacking in Arab countries and Arabs may rely on media, family, and friends as sources of health information and visit a physician only if health problems and symptoms are acute (Donnelly *et al*., 2013).

Here, we also identified concerns about the procedure as a barrier to CRCS. The participants who underwent colonoscopy described it as painful. Physicians should be aware of such concerns among Arab Americans and alternative CRCS methods to colonoscopy should be considered. One of the acceptable alternatives to colonoscopy is FIT, which could be offered to Arab Americans to achieve compliance with CRCS recommendations. Lack of communication with the physician was also found to be a barrier. As the lack of communication affects the physician–patient relationship and leads to less compliance, physicians should receive culturally based training to improve their communication skills with Arab American patients. A multipronged communication program that addresses the barriers identified in our study should be considered to encourage Arab Americans to undergo CRCS.

Three facilitators of CRCS were found as major themes of discourse within our focus groups and interviews. The participants linked their compliance to CRCS because of an interest in prioritizing their health. Access to healthcare was also found to be a second facilitator. Participants who had access to healthcare underwent CRCS and generally followed physicians’ recommendations. From an access perspective, the Affordable Care Act (ACA) allowed millions of Americans to receive cancer preventative services and contributed in reducing CRCS disparities across sensitive populations within our country. Taking advantage of this preventative coverage was also facilitated by awareness. Some participants in our study were largely aware of CRCS through family and friend networks, in addition to their limited physician interactions. As such, health promotion resources and materials can implement the facilitators identified above in an effort to increase the awareness of CRCS among Arab Americans, urging the prioritization of health.

### Limitations

One limitation of our study includes the sample size being small due to low enrollment. Advertisements were left up for at least six months in each location in which the focus groups or interviews were conducted. In addition, only men were recruited, and so, perceptions of CRCS from Arab American women are not presented here. Finally, the majority of participants had health insurance and primary care physicians. Interestingly, however, thematic barriers unrelated to healthcare access were prevalent in our qualitative data.

## Conclusion

The rate of CRCS in Arab Americans is much lower than the national average rate, which indicates the necessity of CRCS education campaigns that specifically target Arab American communities. The formative work described in our pilot study is hypothesis generating; future research directions should focus on directly testing the effect that Arab-American-specific educational resources have on CRCS. Moreover, future health promotion efforts may also consider leveraging our study’s findings when implementing specific interventions to reduce disparities in CRCS among Arab Americans. It is generally the responsibility of the primary care physician to keep his/her patients up to date for all screening tests as primary care physicians are the first in line for following screening guidelines. Here we describe an important reminder to those primary care healthcare providers of their unique opportunity to bridge specific cultural gaps in order to impact screening rates within this at-risk population.

## Appendix 1. Focus group questions

**Table:**

**General questions**
– What is the main source of your health information?
– What comes to your mind first when you hear ‘Colon Cancer’?
– What do you know about colon cancer?
– What do you know about the screening methods of colon cancer?
– Have you ever discussed CRCS methods with your doctor?
**For screened participants**
– How was your experience with the screening method you had before?
– What were the difficulties you encountered during performing the screening?
– What were the factors that made the screening easy to perform?
**For unscreened participants**
– What were the reasons that made you decide not to get screened?
– What might encourage you to get screened?
